# Ultrasound Risk Stratification and Bethesda Cytology for Predicting Malignancy in Thyroid Nodules: A Histopathology-Based Comparative Study

**DOI:** 10.7759/cureus.111403

**Published:** 2026-06-24

**Authors:** Poonam Kujur, Sunil Kumar Mahto, Smita Kumari Gupta, Anshu Jamaiyar

**Affiliations:** 1 Pathology, Rajendra Institute of Medical Sciences, Ranchi, IND

**Keywords:** cytopathology, fine-needle aspiration, histopathology, roc analysis, thyroid neoplasms, thyroid nodules

## Abstract

Background: Thyroid nodules are one of the most frequently seen endocrine conditions in clinical practice and a major challenge in diagnosis, as only a small proportion of them are malignant. The current study aimed to compare the American College of Radiology Thyroid Imaging Reporting and Data System (ACR-TIRADS) ultrasonographic classification with the Bethesda cytological category in thyroid nodules with histopathological diagnosis.

Methods: Our study was conducted as a hospital-based cross-sectional observational analysis in the Departments of Pathology and Radiodiagnosis at Rajendra Institute of Medical Sciences (RIMS), Ranchi. A total of 50 consecutive patients fulfilling the eligibility criteria were included. Ultrasonographic examination was performed using ACR-TIRADS criteria, and fine needle aspiration cytology (FNAC) findings were interpreted according to The Bethesda System for Reporting Thyroid Cytopathology (TBSRTC). Histopathological examination served as the reference standard.

Results: In our study, the mean age was 33.76 ± 12.85 years, and females constituted 34 (68.0%) cases. TIRADS 3 nodules constituted the largest subgroup with 16 (32.0%) cases. Histopathological examination identified 29 (58.0%) benign lesions and 21 (42.0%) malignant lesions. Bethesda cytology demonstrated superior concordance with histopathology, with a specificity of 100% and excellent diagnostic accuracy with an area under the curve (AUC) of 0.81. In contrast, ACR-TIRADS showed poor discriminatory ability with an AUC of 0.51.

Conclusion: Bethesda cytology showed superior concordance and diagnostic accuracy compared with ultrasonographic ACR-TIRADS classification in predicting malignant thyroid nodules. Combined radiological and cytological evaluation may improve diagnostic certainty and support appropriate surgical decision-making.

## Introduction

Thyroid nodules are among the most common endocrine disorders encountered in clinical practice and pose a significant diagnostic challenge because only a small fraction of them are malignant. Palpable thyroid nodules are present in approximately 4-7% of adults, while high-resolution ultrasonography is capable of detecting nodules in up to 68% of the general population, contributing to a substantial increase in incidentally diagnosed thyroid lesions [1]. The incidence of thyroid carcinoma has risen considerably in recent decades, mainly due to advances in imaging techniques and increased detection of papillary thyroid carcinoma at earlier stages [2]. Although the incidence of thyroid carcinoma has increased over the past few decades, the preoperative distinction of benign from malignant nodules continues to be a critical issue in order to avoid unnecessary surgery and morbidity. Because of its non-invasive, widely available, and extremely sensitive characteristics for detecting thyroid lesions, ultrasonography is the major imaging modality used for evaluation of thyroid nodules [3]. The American College of Radiology Thyroid Imaging Reporting and Data System (ACR-TIRADS) is a risk stratification system for thyroid nodules that uses weighted ultrasound characteristics to classify nodules in order of estimated malignancy risk and to recommend for or against fine needle aspiration cytology (FNAC) [4].

FNA is the main investigation used for preoperative evaluation of thyroid nodules due to its simplicity, low cost, high diagnostic accuracy, and thyroid cytology reporting, which has been standardized by The Bethesda System for Reporting Thyroid Cytopathology (TBSRTC) into six categories that are associated with a low risk of malignancy and with clinical management recommendations for category II lesions and a strong correlation of the Bethesda V and VI with malignant histopathology for thyroid [5,6]. Nonetheless, non-diagnostic terms like atypia of undetermined significance and follicular neoplasm remain a challenge to diagnosis due to the inability of cytology to make a judgment on capsular or vascular invasion [7].

However, there is a wide range of reported diagnostic accuracy for ACR-TIRADS in various populations and institutional settings, as several studies have shown a significant correlation between increasing TIRADS categories and increasing Bethesda grades, which indicates that combined radiological and cytological evaluation may be more useful in predicting malignancy [4,8]. The reliability of ultrasound risk stratification remains poor due to interobserver variability, overlapping sonographic signs, and a lack of understanding of follicular-patterned lesions [9]. Furthermore, not much information is available on the level of agreement of ACR-TIRADS with the Bethesda classification in Eastern India, with histopathology as the gold standard [9]. The aim of the current study was to compare the ACR-TIRADS ultrasonographic classification with the Bethesda cytological category in thyroid nodules with histopathological diagnosis. The study also attempted to compare the two techniques in terms of their preoperative diagnostic value and malignancy prediction accuracy. The primary objective of this study was to compare the diagnostic performance of ACR-TIRADS and Bethesda cytology in predicting malignancy in thyroid nodules using histopathology as the reference standard. The secondary objectives were to assess the concordance of each diagnostic system with histopathological findings and to compare their sensitivity, specificity, positive predictive value, negative predictive value, diagnostic accuracy, and ROC performance.

## Materials and methods

Our study was conducted as a hospital-based cross-sectional observational analysis in the Departments of Pathology and Radiodiagnosis at Rajendra Institute of Medical Sciences (RIMS), Ranchi, over a period extending from July 2024 to January 2026 after obtaining approval from the Institutional Ethics Committee. The approval from the Institutional Ethics Committee was received as IEC approval no. 93 dated 01/02/2025, and all participants obtained written informed consent before joining the study. We evaluated patients presenting with clinically apparent thyroid swellings or ultrasonographically detected thyroid nodules who underwent FNAC as part of routine diagnostic workup. Written informed consent was obtained from all participants prior to enrollment.

A total of 50 consecutive patients fulfilling the eligibility criteria were included. The sample size was considered adequate in view of comparable diagnostic concordance studies evaluating TIRADS and Bethesda classification systems in thyroid nodules, supporting the applicability of moderate-sized observational cohorts for diagnostic accuracy assessment [8]. This was a single-center cross-sectional study utilizing all eligible patients who met the inclusion criteria during the study period. No formal sample size calculation was performed, and the study should therefore be considered exploratory. Patients aged between 12 and 55 years with thyroid nodules amenable to ultrasonographic evaluation and FNAC were included. Cases with incomplete clinical records, technically inadequate FNAC, or refusal to participate were excluded from analysis. In our study, ultrasonographic examination of the thyroid gland was performed using a high-frequency linear transducer. Nodules were assessed for composition, echogenicity, shape, margins, and echogenic foci, following the ACR-TIRADS criteria. Nodules were subsequently categorized from TR1 to TR5 according to malignancy risk stratification. In patients with multiple nodules, the lesion demonstrating the highest TIRADS category was selected for evaluation. For diagnostic performance analysis, ACR-TIRADS categories 4 and 5 were considered positive for malignancy, whereas categories 1-3 were considered negative. Similarly, Bethesda categories V and VI were considered positive, whereas categories I-IV were considered negative. 

We performed FNAC using a 22-23 gauge disposable needle under aseptic precautions. Both air-dried and alcohol-fixed smears were prepared and stained with May-Grünwald-Giemsa and Papanicolaou stains, respectively. Cytological interpretation was carried out according to TBSRTC. Histopathological assessment was performed by a single pathologist who was blinded to the study objectives. For diagnostic performance analysis, Bethesda categories V and VI were considered positive for malignancy, whereas Bethesda categories I-IV were considered negative. The distribution of histopathological outcomes across individual Bethesda categories and the corresponding confusion matrix used for diagnostic performance calculations are provided in Appendices 1, 2, respectively. This dichotomization was used to calculate sensitivity, specificity, positive predictive value, negative predictive value, and overall diagnostic accuracy. Histopathological examination of surgically resected specimens served as the reference standard for final diagnosis. Our study included assessment of sensitivity, specificity, positive predictive value, and negative predictive value. Receiver operating characteristic (ROC) curves were generated using histopathological diagnosis as the reference standard. The area under the curve (AUC) was calculated to assess the discriminatory performance of ACR-TIRADS and Bethesda cytology. Statistical analysis was performed using IBM SPSS Statistics for Windows, Version 27 (Released 2019; IBM Corp., Armonk, New York, United States), and a p-value <0.05 was considered statistically significant.

## Results

A total of 50 patients with thyroid nodules were included in our analysis. In our study, the mean age of the study population was 33.76 ± 12.85 years, with the highest proportion of patients belonging to the 31-40-year age group comprising 17 (34.0%) cases. We observed a female predominance, with 34 (68.0%) females and 16 (32.0%) males, resulting in a male-to-female ratio of approximately 1:2.1.

On ultrasonographic evaluation, TIRADS 3 nodules constituted the largest subgroup with 16 (32.0%) cases, followed by TIRADS 4 with 13 (26.0%) cases and TIRADS 5 with 11 (22.0%) cases. In our cytological assessment, Bethesda II represented the most frequent category with 18 (36.0%) cases, whereas Bethesda III and IV together accounted for 18 (36.0%) cases. Histopathological examination in our study identified 29 (58.0%) benign lesions and 21 (42.0%) malignant lesions (Table 1).

**Table 1:** Baseline demographic and diagnostic characteristics of study participants ACR-TIRADS: American College of Radiology Thyroid Imaging Reporting and Data System; TR: TIRADS category

Variable	Category	n (%)
Age group	10–20 Years	10 (20.0%)
	21–30 Years	9 (18.0%)
	31–40 Years	17 (34.0%)
	41–50 Years	8 (16.0%)
	>50 Years	6 (12.0%)
Gender	Female	34 (68.0%)
	Male	16 (32.0%)
ACR-TIRADS category	TR2	10 (20.0%)
	TR3	16 (32.0%)
	TR4	13 (26.0%)
	TR5	11 (22.0%)
Bethesda category	I	3 (6.0%)
	II	18 (36.0%)
	III	8 (16.0%)
	IV	10 (20.0%)
	V	4 (8.0%)
	VI	7 (14.0%)
Histopathology	Benign	29 (58.0%)
	Malignant	21 (42.0%)

We evaluated concordance between ultrasonographic ACR-TIRADS classification, Bethesda cytology, and final histopathological diagnosis. Among histopathologically benign lesions, 14 cases were categorized as malignant by TIRADS, while 11 malignant lesions were incorrectly classified as benign. Our findings demonstrated poor agreement between ACR-TIRADS and histopathology on Cohen’s kappa analysis (κ = -0.006).

In contrast, we observed improved concordance between Bethesda grading and histopathological diagnosis. All 29 (100%) histopathologically benign lesions were categorized within benign Bethesda groups, whereas 11 malignant lesions were classified within the Bethesda malignant categories. Moderate agreement was observed between Bethesda grading and histopathology (κ = 0.56) (Table 2).

**Table 2:** Concordance of ACR-TIRADS and Bethesda grading with histopathological diagnosis ACR-TIRADS: American College of Radiology Thyroid Imaging Reporting and Data System; κ: Cohen's kappa coefficient

Diagnostic modality	Histopathology benign n (%)	Histopathology malignant n (%)	Cohen’s κ
TIRADS benign	15 (51.7%)	11 (52.4%)	–0.006
TIRADS malignant	14 (48.3%)	10 (47.6%)	
Bethesda benign	29 (100%)	10 (47.6%)	0.56
Bethesda malignant	0 (0%)	11 (52.4%)	

Our study further demonstrated the superior diagnostic performance of the Bethesda system compared with ACR-TIRADS. Ultrasonographic TIRADS classification showed sensitivity and specificity of 47.6% and 51.7%, respectively, with a positive predictive value of 41.7% and a negative predictive value of 57.7%. In comparison, Bethesda cytology demonstrated a specificity and positive predictive value of 100%, along with an improved negative predictive value of 74.4% (Table 3). No benign lesion was classified as Bethesda category V or VI in the present cohort (Appendix 2), resulting in a specificity and positive predictive value of 100%. 

**Table 3:** Diagnostic performance of ACR-TIRADS and the Bethesda system ACR-TIRADS: American College of Radiology Thyroid Imaging Reporting and Data System; CI: confidence interval

Parameter	ACR-TIRADS (95% CI)	Bethesda system (95% CI)
Sensitivity	47.6% (25.7–70.2%)	52.4% (29.8–74.3%)
Specificity	51.7% (32.5–70.6%)	100.0% (88.1–100.0%)
Positive Predictive Value	41.7% (22.1–63.4%)	100.0% (71.5–100.0%)
Negative Predictive Value	57.7% (36.9–76.6%)	74.4% (57.9–87.0%)

AUC analysis in our study revealed poor discriminatory ability for ACR-TIRADS, with an AUC of 0.51. In contrast, the Bethesda system showed excellent diagnostic accuracy with an AUC of 0.81 for predicting malignant lesions (Table 4).

**Table 4:** AUC characteristic analysis of diagnostic modalities ACR-TIRADS: American College of Radiology Thyroid Imaging Reporting and Data System; AUC: area under the curve; CI: confidence interval

Diagnostic modality	AUC	p-value	95% CI	Interpretation
ACR-TIRADS	0.51	0.271	0.407-0.749	Poor discriminatory ability
Bethesda system	0.81	0.001	0.727-0.899	Excellent diagnostic accuracy

The ROC curve for the Bethesda system covers a larger area and stays closer to the left upper corner than that of the ROC curve for the TIRADS system, showing the better discriminative accuracy of the Bethesda system. The malignancy risk by TIRADS and Bethesda categories was also evaluated. There was no difference in the malignancy risk between nodules classified as TIRADS 2-3 and TIRADS 4-5 in our study (42.31% and 41.67%, respectively), or in the number of malignant cases among the nodules classified as TIRADS 2-3 and 4-5 (11 and 10, respectively).

By comparison, malignancy risk was seen to increase progressively with Bethesda categories. The Bethesda III-IV category showed intermediate malignancy risk, with no malignant lesion found in the Bethesda I-II groups. Pathologically, all 11 (100%) Bethesda V-VI nodules showed malignancy (Table 5).

**Table 5:** Risk of malignancy according to ACR-TIRADS and Bethesda categories ACR-TIRADS: American College of Radiology Thyroid Imaging Reporting and Data System; TR: TIRADS category

Classification system	Category	Total cases (n)	Malignant cases (n)	Risk of malignancy (%)
ACR-TIRADS	TR2–TR3	26	11	42.31%
	TR4–TR5	24	10	41.67%
Bethesda system	I–II	21	0	0.00%
	III–IV	18	10	55.56%
	V–VI	11	11	100.00%

Histopathological outcomes for each individual Bethesda category are summarized in Appendix 1. Overall, our study demonstrated that Bethesda cytology showed superior concordance and diagnostic accuracy compared with ultrasonographic ACR-TIRADS classification in predicting malignant thyroid nodules. The confusion matrix used for the calculation of these diagnostic performance metrics is presented in Appendix 2.

## Discussion

Our overall malignancy rate of 21 (42%) was higher than that reported in population-based studies [1,10], which may be due to referral bias and the possibility of a higher proportion of clinically suspicious and cytologically indeterminate nodules being operated on in a tertiary care setting. Similar observations were reported by Haugen et al., who noted increased malignancy prevalence in institutional surgical cohorts because of selective referral patterns [11]. The mean age of the patients was 33.76 ± 12.85 years, and the majority of patients were in the age group 31-40 years, comprising 17 (34%) cases. Biswas et al. and Gietka M reported that the predominance of women with thyroid nodules is similar to that reported in other studies, and may be explained by hormonal factors and autoimmune thyroid disease [12,13]. However, the age profile of our cohort differed from the studies by Sreeram et al. and De Fiori et al., who reported mean ages above 40 years [14,15]. Regional demographic differences and healthcare-seeking behavior may explain this variation.

Among the ultrasonographic TIRADS categories, TIRADS 3 was the most prevalent with 16 (32%) nodules, followed by TIRADS 4 with 13 (26%) nodules and TIRADS 5 with 11 (22%) nodules. Concordance between ACR-TIRADS and histopathology was poor, with a Cohen’s kappa coefficient of −0.006, and ROC analysis demonstrated limited discriminatory ability with an area under the curve of 0.51. These findings differ substantially from previous studies by Tessler et al., Patro et al., and Kwak et al., which reported increasing malignancy risk across higher TIRADS categories and better diagnostic performance [4,8,16]. Several factors may explain this discrepancy, including the relatively small sample size, referral bias, operator dependency of ultrasonography, interobserver variability in sonographic interpretation, and institution-specific implementation of TIRADS criteria. Hoang et al. similarly demonstrated variability in the assessment of several ultrasound features used in TIRADS classification [17]. Since interobserver agreement and radiologist-specific performance were not evaluated in the present study, the observed findings should be interpreted as institution-specific observations rather than evidence against the overall validity of the ACR-TIRADS system.

In contrast, Bethesda cytology demonstrated superior concordance with histopathology, with moderate agreement (κ = 0.56), specificity of 100%, and excellent diagnostic performance (AUC = 0.81). These findings are consistent with studies by Cibas and Ali and by Bongiovanni et al., which highlighted the reliability and reproducibility of the Bethesda system in malignancy risk assessment [6,18]. We also observed a progressive increase in malignancy risk across higher Bethesda categories, with all 11 (100%) Bethesda V-VI lesions proving malignant on histopathology, similar to observations reported by Ilyas et al. [19]. Overall, our findings suggest that Bethesda cytology provides superior diagnostic accuracy and concordance compared with ACR-TIRADS in predicting malignant thyroid nodules. Although ultrasonography is useful in the initial risk stratification and selection of nodules for FNAC, cytology still has a major role in the definitive preoperative assessment. Histopathology was used as a reference standard, and both diagnostic systems were compared, enhancing our analysis.

Several limitations should be considered when interpreting our findings. First, the relatively small sample size and limited number of malignant cases resulted in wide confidence intervals and reduced statistical precision. Second, the single-center design may have introduced referral and selection bias, thereby limiting the generalizability of the findings. Third, interobserver variability among radiologists was not assessed, and details regarding operator experience were not available for analysis. Fourth, molecular testing was not performed, which may have provided additional diagnostic information, particularly for indeterminate Bethesda categories. Future multicenter studies with larger sample sizes, standardized ultrasonographic assessment, evaluation of interobserver agreement, and integration of molecular markers are needed to further validate and refine the comparative diagnostic performance.

## Conclusions

In our single-center study, Bethesda cytology demonstrated greater concordance with histopathology and higher diagnostic accuracy than ACR-TIRADS classification for predicting malignant thyroid nodules. However, the diagnostic performance of ACR-TIRADS observed in our study was substantially lower than that reported in previous literature and should be interpreted cautiously, given the relatively small sample size and potential institution-specific factors. Ultrasonography remains an important tool for initial risk stratification and selection of nodules for FNAC, whereas cytological evaluation continues to play a central role in preoperative assessment. Larger multicenter studies with standardized ultrasonographic assessment and evaluation of interobserver variability are required to further clarify the comparative performance of these diagnostic systems.

## Appendices

Appendix 1 

**Table 6:** Histopathological outcomes according to individual Bethesda categories ROM: risk of malignancy

Bethesda category	Benign	Malignant	Total	ROM (%)
I	3	0	3	0.0
II	18	0	18	0.0
III	3	5	8	62.5
IV	5	5	10	50.0
V	0	4	4	100.0
VI	0	7	7	100.0
Total	29	21	50	42.0

Appendix 2

**Table 7:** Confusion matrix for the Bethesda system

Bethesda result	Malignant histology	Benign histology
Positive (V–VI)	11	0
Negative (I–IV)	10	29
